# Semi-field assessment of the BG-Malaria trap for monitoring the African malaria vector, *Anopheles arabiensis*

**DOI:** 10.1371/journal.pone.0186696

**Published:** 2017-10-18

**Authors:** Elis P. A. Batista, Halfan S. Ngowo, Mercy Opiyo, Gasper K. Shubis, Felician C. Meza, Fredros O. Okumu, Alvaro E. Eiras

**Affiliations:** 1 Laboratório de Ecologia Química de Insetos Vetores, Departamento de Parasitologia, Instituto de Ciências Biológicas, Universidade Federal de Minas Gerais, Belo Horizonte, Brazil; 2 Environmental Health and Ecological Sciences Department, Ifakara Health Institute, Ifakara, Tanzania; 3 School of Public Health, Faculty of Health Sciences, University of the Witwatersrand, Parktown, Republic of South Africa; 4 Institute of Biodiversity, Animal Health and Comparative Medicine, University of Glasgow, Glasgow, United Kingdom; Johns Hopkins University, UNITED STATES

## Abstract

Odour-baited technologies are increasingly considered for effective monitoring of mosquito populations and for the evaluation of vector control interventions. The BG-Malaria trap (BGM), which is an upside-down variant of the widely used BG-Sentinel trap (BGS), has been demonstrated to be effective to sample the Brazilian malaria vector, *Anopheles darlingi*. We evaluated the BGM as an improved method for sampling the African malaria vectors, *Anopheles arabiensis*. Experiments were conducted inside a large semi-field cage to compare trapping efficiencies of BGM and BGS traps, both baited with the synthetic attractant, Ifakara blend, supplemented with CO_2_. We then compared BGMs baited with either of four synthetic mosquito lures, Ifakara blend, Mbita blend, BG-lure or CO_2_, and an unbaited BGM. Lastly, we compared BGMs baited with the Ifakara blend dispensed via either nylon strips, BG cartridges (attractant-infused microcapsules encased in cylindrical plastic cartridge) or BG sachets (attractant-infused microcapsules encased in plastic sachets). All tests were conducted between 6P.M. and 7A.M., with 200–600 laboratory-reared *An*. *arabiensis* released nightly in the test chamber. The median number of *An*. *arabiensis* caught by the BGM per night was 83, IQR:(73.5–97.75), demonstrating clear superiority over BGS (median catch = 32.5 (25.25–37.5)). Compared to unbaited controls, BGMs baited with Mbita blend caught most mosquitoes (45 (29.5–70.25)), followed by BGMs baited with CO_2_ (42.5 (27.5–64)), Ifakara blend (31 (9.25–41.25)) and BG lure (16 (4–22)). BGM caught 51 (29.5–72.25) mosquitoes/night, when the attractants were dispensed using BG-Cartridges, compared to BG-Sachet (29.5 (24.75–40.5)), and nylon strips (27 (19.25–38.25)), in all cases being significantly superior to unbaited controls (p < 000.1). The findings demonstrate potential of the BGM as a sampling tool for African malaria vectors over the standard BGS trap. Its efficacy can be optimized by selecting appropriate odour baits and odour-dispensing systems.

## Introduction

Large-scale implementation of the two front-line vector control interventions against African malaria vectors, i.e., long-lasting insecticidal nets (LLINs) and indoor residual spraying (IRS), have led to major reductions in malaria cases contributing just over three quarters of all gains since 2000 [1]. Despite these gains, there appears to be persistent transmission, a significant proportion of which may be occurring outdoors and is not targeted effectively by LLINs and IRS [2, 3]. Other challenges include the growing physiological insecticide resistance in vector populations [4–6] and behavioural responses of the residual malaria vectors, which may also lower their responsiveness to control [7–9]. These challenges, combined with poor user-compliance and human behaviours, such as spending most of the time outside dwellings in the evenings, heavily compromise the likelihood of malaria elimination in many settings [10–12].

An important and closely related problem is the need for new tools to monitor this persistent transmission, especially in areas where a significant proportion of biting occurs outdoors [7, 13, 14], but also in areas where specific interventions need to be matched to certain vector species behaviours and responsiveness. Odour-baited devices have been proposed as potential complementary tools to sample malaria mosquitoes outdoors [15–21], but also to disrupt transmission [22]. Examples of odour-baited traps or human-baited traps previously used for malaria mosquitoes include, the Suna Trap [23], Odour-Baited Mosquito Entry Trap [20, 24], Ifakara-Tent Trap [20], Ifakara Odour-baited stations [25], the MMX trap [26, 27], the Mosquito Landing Box [18] and BG-Sentinel Trap [28].

The BG-Malaria trap (BGM) [29, 30] is a modified version of the original BG-Sentinel (BGS) trap created by Biogents Company, Germany. The BGM trap was adapted to collect *Anopheles* species. BGM has already been demonstrated as a sensitive method for monitoring Brazilian malaria vectors [29, 30], but it has not previously been tested for African malaria vectors. The main difference between the two traps is the airflow orientation. The BGM is installed upside down, 40 cm above the ground, making it a simple adaptation of BGS. This adaptation was considerably more effective than other commercial traps tested, including the original BGS and CDC-Light Traps, and performed almost as well as human landing catches (HLC) in Brazilian field tests [29].

In the initial trials, the bait used in BGM trap was CO_2_ obtained from dry ice [29, 30]. However, to improve trap efficacy, other baits such as synthetic human odours should also be explored. The synthetic odour blend developed at Ifakara Health Institute (i.e. Ifakara Blend) is one candidate lure, already demonstrated in long-range village tests to be more attractive to malaria and non-malaria mosquitoes, than real humans [31]. There are several other synthetic attractant variants, potentially equal or perhaps more effective.

Other than the actual mosquito attractants, studies also have demonstrated that performance of these attractants is influenced by the medium from which they are dispensed [32–34]. For example, nylon strips have been used effectively for the release of attractants for host-seeking malaria mosquitoes [31, 33–38]. Other examples of odour dispensing materials have included low density polyethylene (LDPE) sachets [39–41] and glass vials [31, 33]. These have shown that an appropriate selection of the dispensing medium to liberate the odorants should be considered at least as important as the actual attractants or traps.

The main aim of this study was to evaluate the efficiency of an odour-baited BGM trap for sampling host-seeking laboratory-reared *An*. *arabiensis*. We examined the efficacy of the BGM relative to the original BGS trap and compared four different attractant types for baiting the BGM. We also tested three different odour-dispensing methods used in the BGM.

## Materials and methods

### Study site

The work was conducted at Ifakara Health Institute, in a semi-field system facility [42] located at Ifakara branch, in Kilombero district, southeastern Tanzania. The semi-field system consists of three-chambered large screened-enclosures, measuring 28.8m by 21m, with walls made of UV-resistant shade netting, and a polyethylene roof mounted on a raised concrete platform [42]. We used two chambers of this facility, each measuring 28.8m by 7m.

### Mosquitoes

Laboratory-reared *An*. *arabiensis* mosquitoes were used. The colony (with mosquitoes originally from Lupiro village, Ulanga district, Tanzania) has been maintained in the laboratory at Ifakara Health Institute since 2009. The mosquitoe larvae are reared under standard insectary conditions (29±1°C, 80±5% RH and 12:12h photoperiod) and fed with Tetramin^®^ fish food. Adult mosquitoes were kept in a separate room, where temperatures were maintained at an average temperature of ~27°C and relative humidity at 70–90%. Female and male adults were housed together in a 30 x 30 x 30 cm mating cage and 10% sucrose solution was placed in the cage as a food source. To propagate the colony, the adult female mosquitoes were fed also on human blood (by way of volunteer arm-feeding from five to ten mins every two days). *An*. *arabiensis* females aged three to eight days that had not previously taken any blood meals were used for this study. Before beginning of the experiments each night, mosquitoes were selected early and starved by withdrawing the glucose solution, 6 h in advance, to encourage host-seeking behaviour.

### BG-Sentinel trap (BGS) and BG-Malaria trap (BGM)

The BGS (BioGents HmGb, Regensburg, Germany) has a cylindrical shape, 35cm in diameter and 40cm in height. Centered inside the trap is a black collecting tube (12cm in diameter x 30cm in length), with a bag for collecting mosquitoes. An electric fan (12V, 14 cm diameter, powered by a 12 V battery) produces a cycle downward flow of air that exits through a gauze cover on the top of the trap and draws in mosquitoes that approach the collecting tube [28]. The interior of the trap can be fitted with different types of mosquito attractants, from which the odours exit through the gauze cover to lure mosquitoes [28].

**Fig 1:**
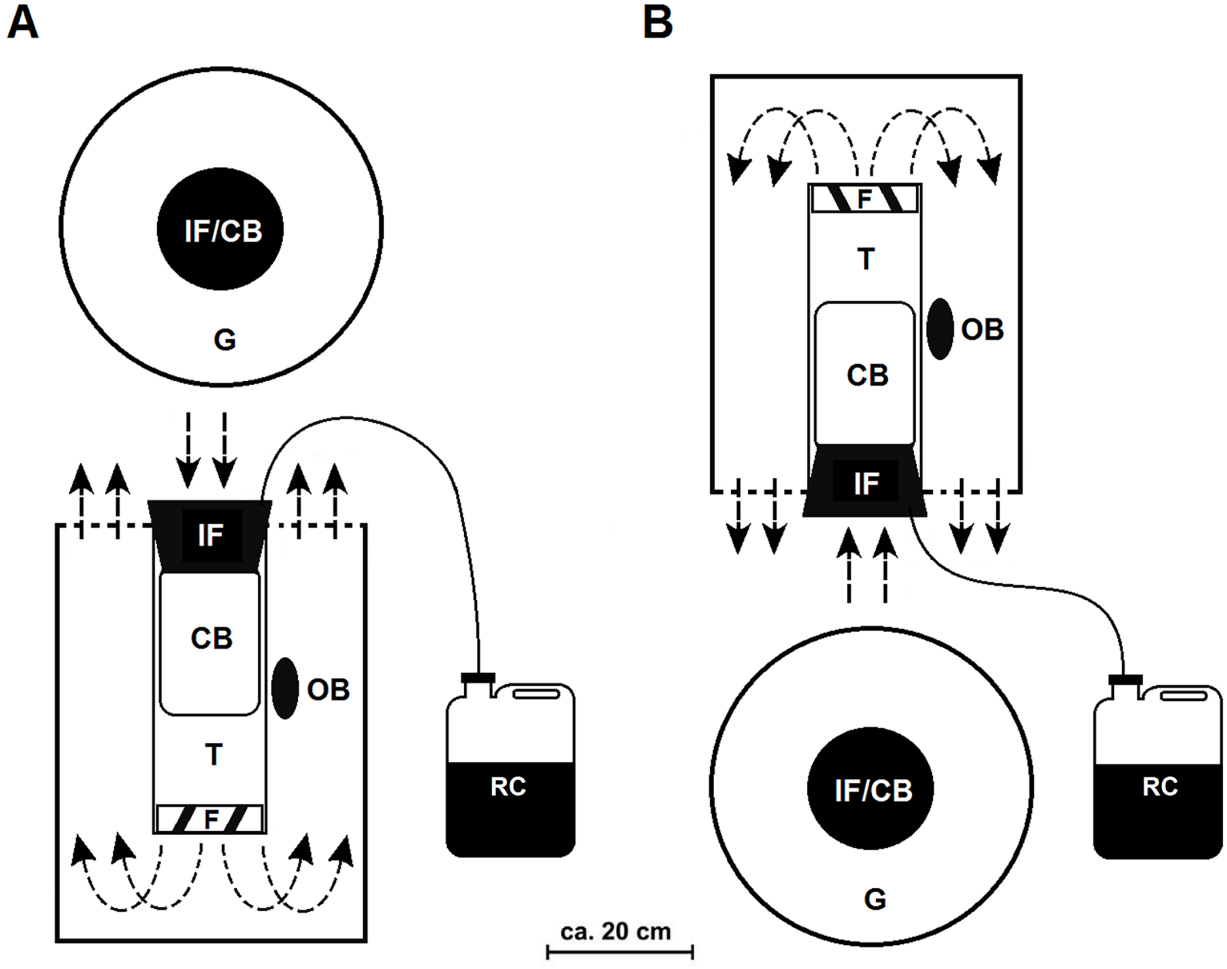
Illustration of design and functionality of: (A) BG-Sentinel and (B) BG-Malaria. IF = Intake funnel; CB = Catch Bag; F = Fan; G = Gauze Cover; T = Tube; RC = Recipient of CO_2_; OB = Odour Bait. Arrows indicate the direction of the airflow. Adapted from Kröckel *et al*., (2006) and Gama *et al*., (2013).

The BGM is an upside down variant of the BGS [29]. The BGM is hanged upside down, 40cm above the ground, with an electrical fan (12V, 14cm diameter, powered by a 12V battery), which produces an upward suction to capture the mosquitoes that approach the trap. The two traps, BGS and BGM, are illustrated in Fig 1.

### Synthetic attractants

Three synthetic mosquito attractants were used: **(i)** the BG-Lure (BioGents HmGb, Regensburg, Germany), which consists of a mixture of ammonia, L-lactic acid, and caproic acid, in undeclared proprietary concentrations; **(ii)** the Mbita-5 lure (MB5), which consists of ammonia (2.5%), lactic acid (85%), tetradecanoic acid (0.00025%) and 3-methyl-1-butanol (0.000001%) [38]; **(iii)** the Ifakara blend (IB), which consists of a mixture of ammonia (2.5%), L-lactic acid (85%), propionic acid (0.1%), butanoic acid (1%), pentanoic acid (0.01%), 3-methylbutanoic acid (0.001%), heptanoic acid (0.01%), octanoic acid (0.01%) and tetradecanoic acid (0.01%) [31]. The IB was used in all the experiments in this work, unlike the BG-Lure and MB5, which were used only in the second experiment.

### Attractant dispensing systems

**Fig 2:**
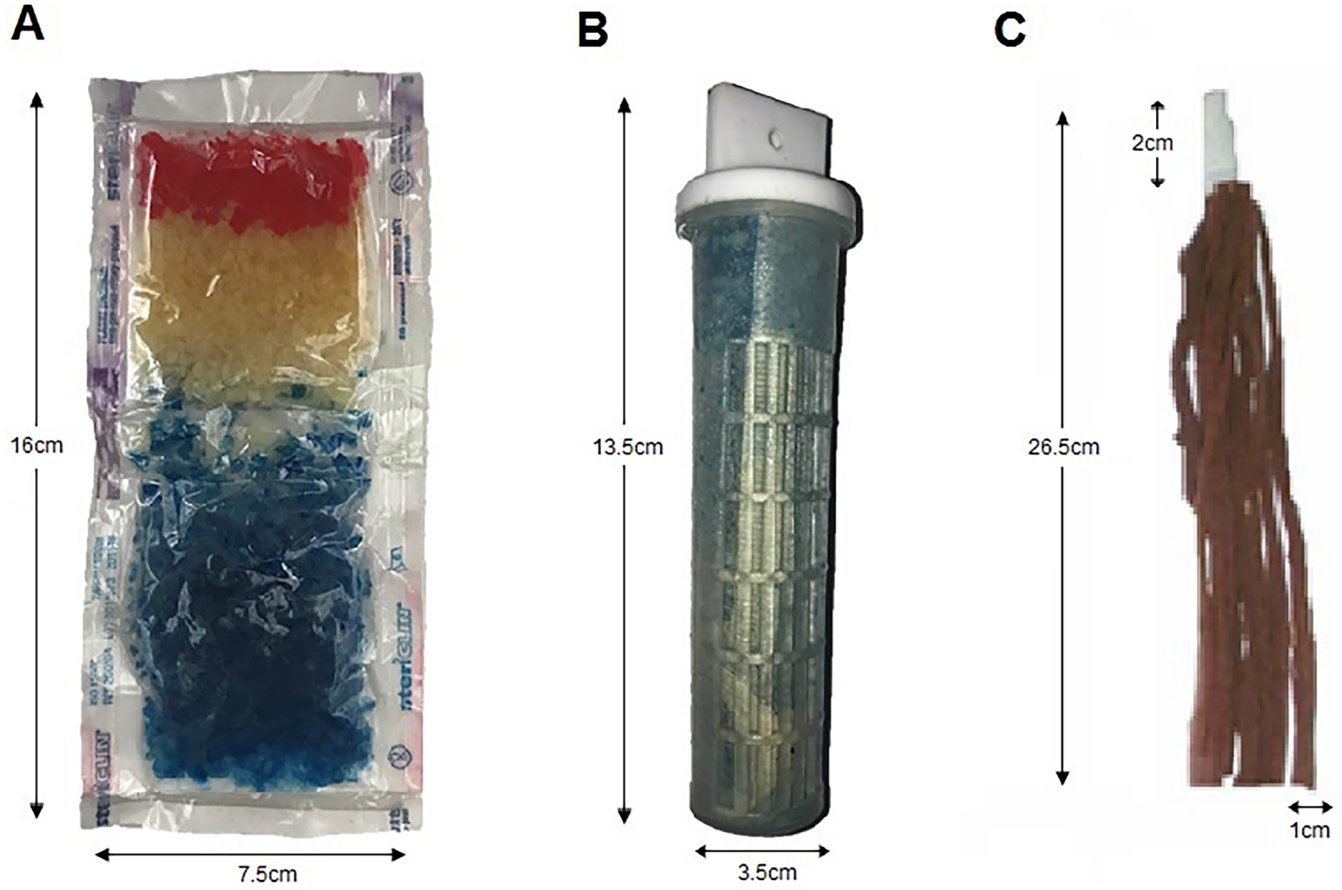
Devices used for dispensing mosquito attractants. Panels A and B show attractants infused inside microcapsules supplied by Biogents Company encased in a plastic sachet (BG-Sachet) and plastic cartridge (BG-Cartridge), respectively. Panel C shows a batch of nylon strips, each soaked in solution of a different constituent of the synthetic attractant [33].

To dispense the synthetic attractants in all the experiments, microcapsules incased in a plastic cartridge (here referred to as BG-Cartridge) supplied by Biogents Company was used. For the experiment where odour-dispensing systems were tested, two others odour-release devices were added. The first was nylon strips, initially tested for dispensing odours attractive to *An*. *gambiae* in Tanzania [33]. The strips are small pieces of nylon, each measuring 26.5 x 1.0 cm, and infused with different attractant chemicals that make up the attractant blend. To make up the IB, nine of these strips are batched together (each strip carrying a different blend constituent) and then suspended inside the candidate mosquito trap. The second dispensing system added was the BG-Sachet (also supplied by Biogents Company), which consisted of microcapsules of the IB odorant constituents encased inside a thin plastic sachet. The individual dispensing methods are illustrated in Fig 2.

### Study procedures

The study consisted of three experiments, conducted nightly between 06.00 P.M. and 07.00 A.M.

#### Experiment 1

**Fig 3:**
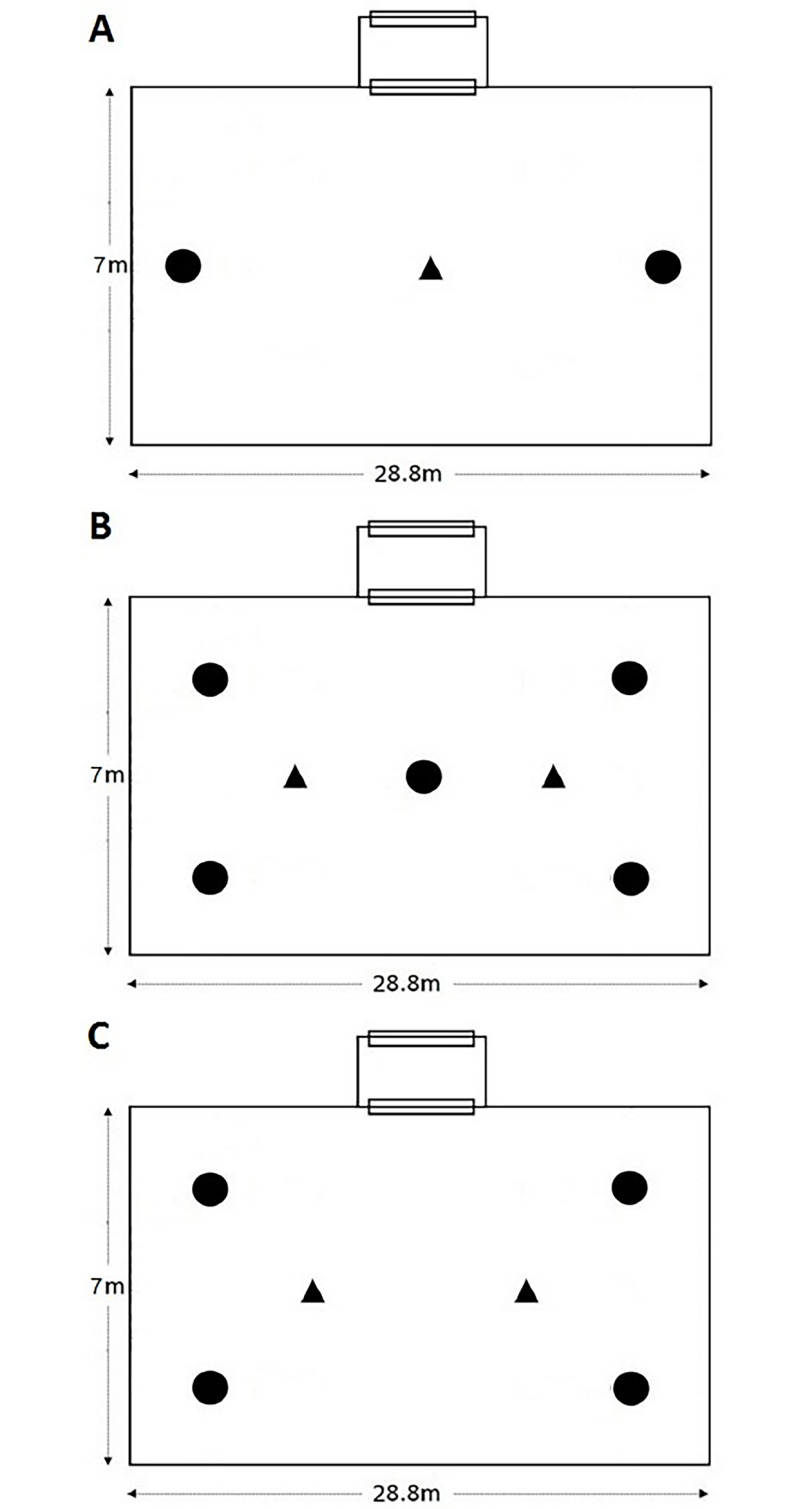
Schematic diagram of the trap positions and mosquito release points within the semi-field system. Set ups for experiments 1, 2 and 3, are shown in figure panels A, B and C, respectively. Trap positions are shown in circles, and mosquito release points in triangles. In all experiments, the treatment being tested was rotated between the test locations nightly.

A binary choice experiment (Fig 3A) was conducted to compare BGM against BGS for catching host-seeking *An*. *arabiensis* mosquitoes. The traps were baited with the synthetic human odour developed at Ifakara Health Institute [31], supplemented with CO_2_ gas obtained from yeast-molasses fermentation. Each night, the BGM or BGS were placed at opposite ends of the semi-field testing chamber. This experiment was replicated 10 times, each time releasing 200 adult female *An*. *arabiensis* mosquitoes at the center of the test chamber (Fig 3A). Each night the trap locations were interchanged to annul any position-related biases. The traps were both powered by 12V rechargeable batteries. The experiment was left to run overnight. The traps were emptied each morning, and all mosquitoes caught in each trap counted and recorded.

#### Experiment 2

A comparative evaluation was conducted to compare different candidate lures and identify the most effective one for use with the BGM trap for capturing host-seeking *An*. *arabiensis*. A 5 x 5 Latin square experiment with four replicates was carried out. Four BGM traps were baited with either of the four candidate lures (Fig 3B), and a fifth BGM was un-baited. In many field and laboratory studies, synthetic mosquito attractants are commonly augmented with CO_2_, to activate the mosquitoes and synergize attractive effects of the lures. We maintained this practice but also had a separate configuration where CO_2_ was the only bait, to improve comparison of the lures and separate effects of the CO_2_. The five different treatments compared in this experiment were (i) Ifakara blend + CO_2_ (referred to simply as IB); (ii) BG-Lure^®^ + CO_2_; (iii) Mbita-5 Blend + CO_2_ (referred to as MB5); (iv) CO_2_ alone; and (v) a control consisting of non-baited BGM trap. The IB, MB5 and BG-Lure were dispensed using BG-Cartridges supplied by Biogents Company. The CO_2_ was obtained from a mix of 2L water with 500g molasses plus 35g yeast, delivered from a 5L plastic container, through a 60 cm long plastic tubing measuring 0.5 cm in diameter [43].

For each night’s experiment, 600 host-seeking female *An*. *arabiensis* mosquitoes were released in the semi-field chamber. In this experiment, we used two different release points as illustrated in Fig 3B, with 300 mosquitoes released from each point. The locations of the baits were rotated nightly such that after five nights of experimentation, each bait had been to each location at least once. The experiments ran the entire night, and the traps were emptied in the morning, after which number of mosquitoes collected in each trap was recorded for each treatment. The traps were then thoroughly cleaned using a solution of 70% alcohol and dried outdoors before they could be re-used. The experiment was replicated four times over a 20 nights experimental period.

#### Experiment 3

In the third experiment, we aimed to assess whether the attractiveness of the Ifakara Blend [31] would be affected by the medium from which it is released when used in the BGM supplemented with CO_2_ from yeast-molasses fermentation. The dispensing devices tested are shown in Fig 2 and were as follows: (i) Nylon strips [33]; (ii) BG-Sachet (iii) BG-Cartridge; and (iv) control (no baited trap). The nylon strips were freshly prepared for this experiment, while both the sachets and cartridges were provided by the manufacturer, Biogents Company.

The dispensing devices were rotated in four positions during four nights in a 4 x 4 Latin square design, such that after a complete set, each dispensing method had been to each of the four selected locations at least once. The locations of the traps are illustrated in Fig 3C. We marked and fixed the individual trap locations, but locations of the lures were rotated nightly in a random fashion. The experiment was replicated 5 times, over a 20 nights experimental period. Each night, a total of 400 host-seeking *An*. *arabiensis* mosquitoes were released from two different points in the semi-field test chamber (200 mosquitoes per release point). The number of mosquitoes collected in each trap was recorded each morning.

### Data analysis

The analysis was done using R software version 3.3.2 [44]. In all experiments, Generalized Linear Mixed Effects Statistical Models (GLMMs) were used to estimate the number of mosquitoes captured as a function of the different trap types, dispensing devices or lures. To account for the over-dispersion, number of mosquitoes captured (i.e., the mosquito count data) were modeled following a negative binomial distribution with log link function [45]. In the first experiment the main effects were trap type. In the second experiment (testing the effect of different lures on the number of mosquito captured), the main effect was type of lure. In the last experiment (testing the effect of different odour dispensing mechanism), the main effect was type of dispensing devices. To account for the variation in temperatures, winds and any other confounding factors during the study period, the experiment date, replication number and trap locations were treated as random factors for each analysis in respective experiments. Relative risks (RR), and 95% Confidence Intervals (CI) were used to estimate the strength of influence of each main factor. The estimates were considered statistically significant different if p < 0.05. Additionally, pairwise comparison tests were done using Tukey’s honest significance difference post-hoc test (Tukey’s HSD) to assess differences between individual groups.

### Ethical considerations

A written and signed informed consent was obtained from the volunteers working in the mosquito-rearing facility, and arm-feeding of mosquitoes in the insectary was done only by volunteering adult males. This study was approved by both Ifakara Health Institute IRB (IHI/IRB/No: 34–2014) and the Medical Research Coordinating Council at the Tanzania National Institute of Medical Research (Certificate No. NIMR/HQ/R.8a/Vol.IX/1903).

## Results

### Experiment 1: Test to compare BG Malaria and BG Sentinel traps

**Fig 4:**
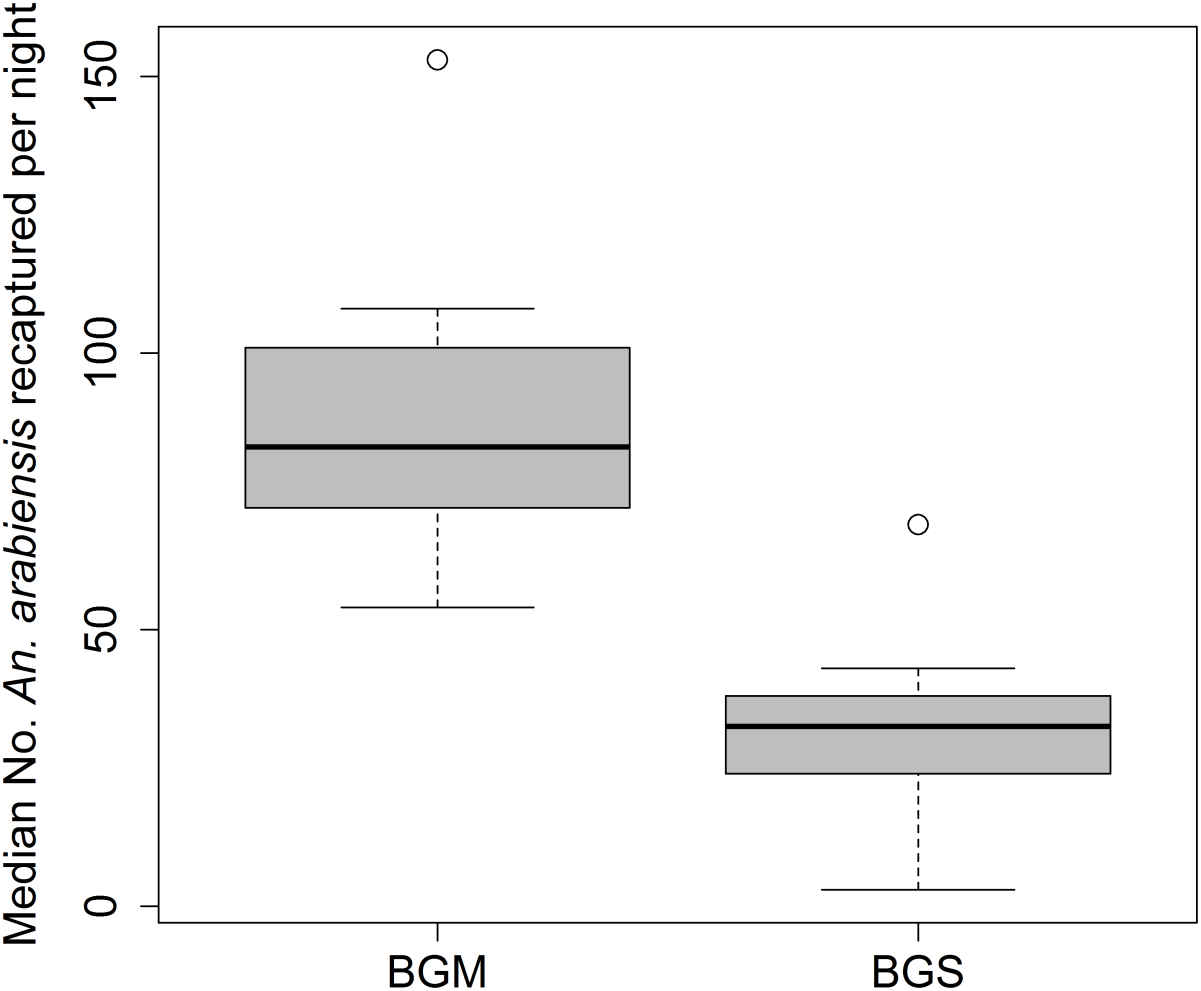
Distribution and median number of *Anopheles arabiensis* recaptured per night using different trapping methods in the semi-field system. BGM = BG-Malaria trap; BGS = BG-Sentinel trap.

Of the 2000 mosquitoes released in the semi-field system chambers throughout the study period, 60.8% were recaptured by the traps. In this experiment, the BGM trap was tested against the BGS trap in a binary choice assay and both traps baited with IB and CO_2_. The median nightly catches are shown in Fig 4. The BGM trap was the more effective trap in capturing mosquitoes [RR = 2.76, 95%CI: (1.95–3.89), p < 0.001] as compared to BGS trap. There was no effect of location on the overall mosquito catches between chambers. Location was not found to have any significant impact on the number of mosquito collected per trap (p = 0.773).

### Experiment 2: Test to compare the different mosquito attractants

**Fig 5:**
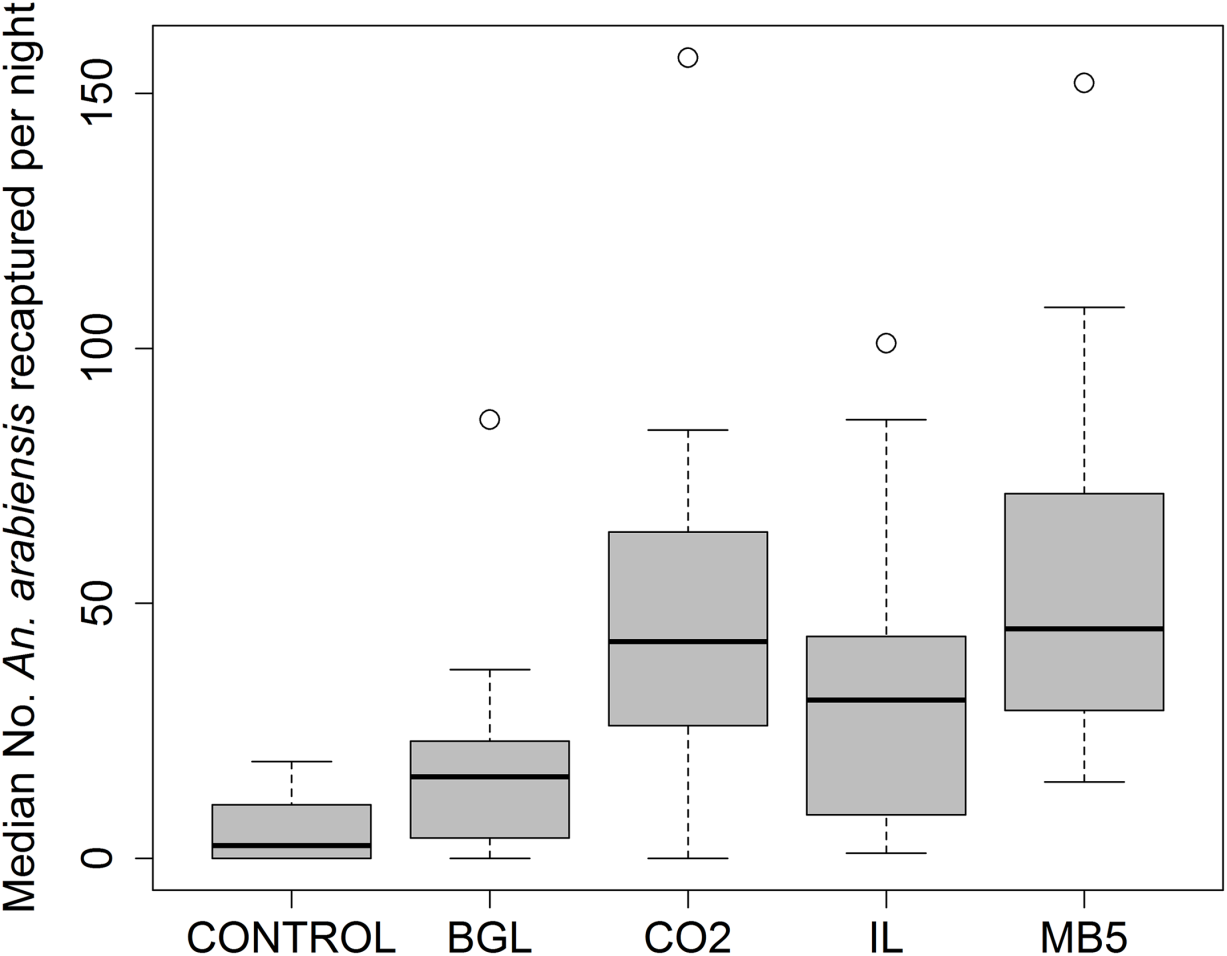
Distribution and median number of *Anopheles arabiensis* recaptured per night by BGM traps baited with different lures in the semi-field system. CO_2_ = Trap baited with only CO_2_; MB5 = Trap baited with Mbita lure + CO_2_; IB = Trap baited with Ifakara blend + CO_2_; BG = Trap baited with BG-Lure + CO_2_; Control = unbaited trap.

A total of 15000 mosquitoes were used in this experiment, for which 20.8% of the total were re-captured in the traps. The number of mosquitoes caught was influenced significantly by type of attractant used (p < 0.001; DF = 4). While traps baited with MB5, IB and CO_2_ alone caught statistically similar numbers of mosquitoes, these were all significantly higher than the number caught in traps baited with the BG-Lure (p < 0.001) or the unbaited traps (p < 0.001). The median numbers of mosquitoes caught in traps baited by different lures is shown in Fig 5.

**Fig 6:**
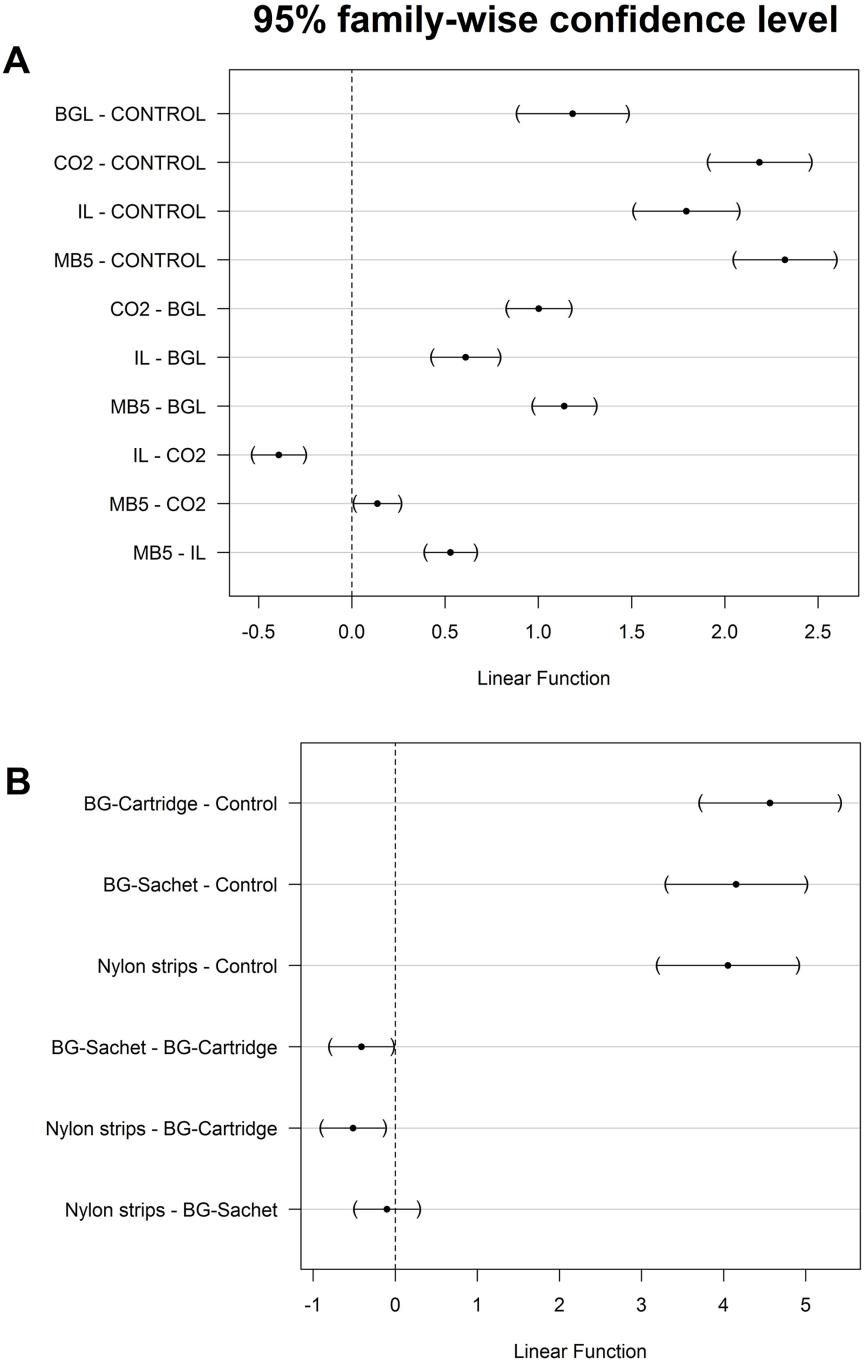
Results of pair-wise post hoc comparison using Tukey’s honestly significance tests (Tukey’s HSD). Howing similarities and differences between number of mosquitoes caught in traps baited with different lures (Panel A) and number of mosquitoes caught in traps baited with different lures dispensed from different media (Panel B).

As compared to the un-baited BGM, traps baited with MB5 caught the highest number of mosquitoes [RR = 10.50, 95%CI: (5.80–18.99), p < 0.001], followed by traps baited with CO_2_ [RR = 8.70, 95%CI: (4.81–15.75), p < 0.001], IB [RR = 6.34, 95%CI: (3.48–11.58), p < 0.001], and BG-Lure [RR = 3.36, 95%CI: (1.84–8.38), p < 0.001]. Pair-wise comparison test using Tukey’s HSD showed that, there is significant difference between CO_2_ and BG-Lure (z = 0.29, p < 0.05), MB5 and BG-Lure (z = 0.29, p < 0.001) while the rest of the pairs were not significantly different from one another (Fig 6A).

### Experiment 3: Test to compare different odour-dispensing methods

**Fig 7:**
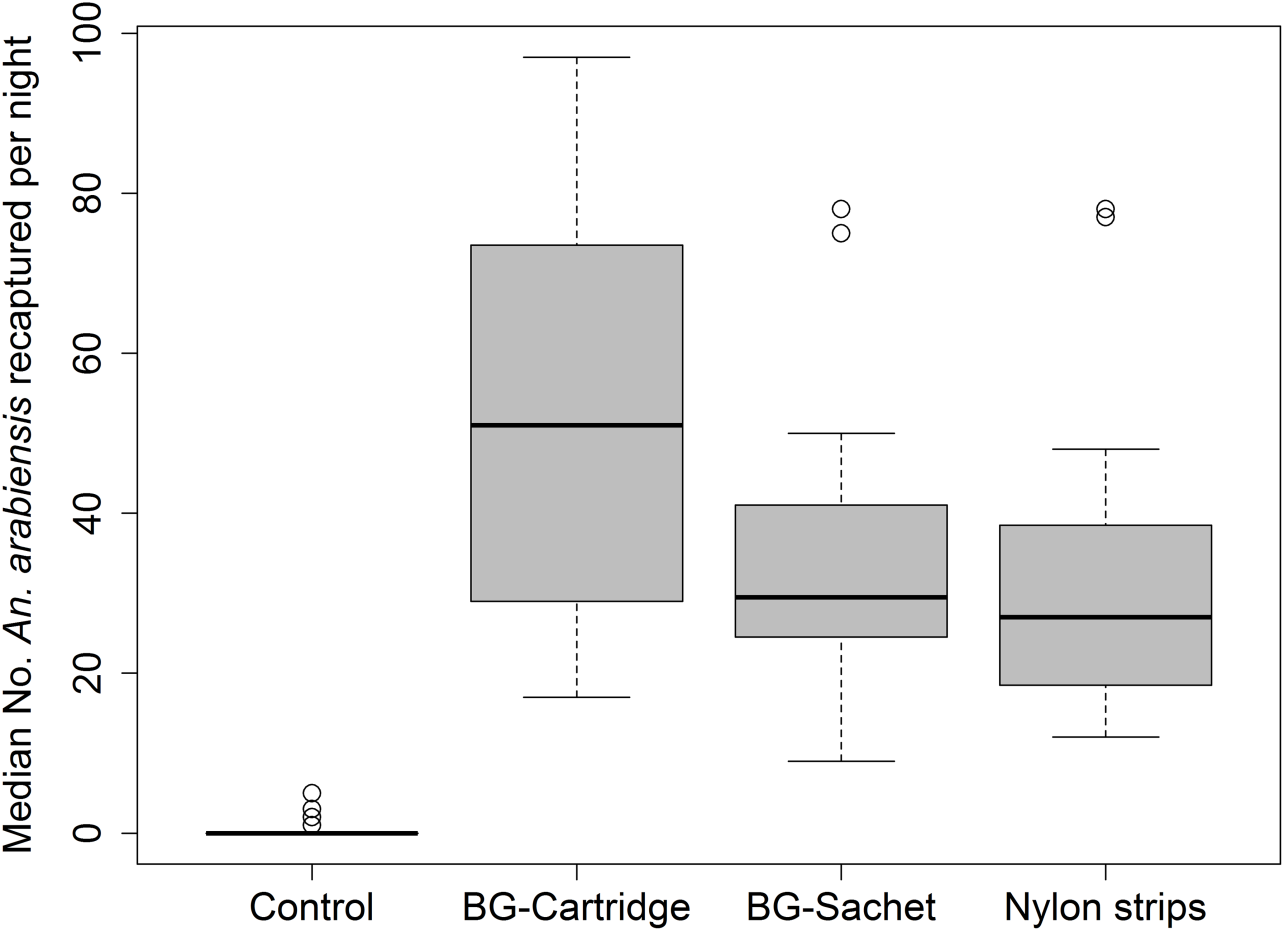
Distribution and median number of *Anopheles arabiensis* recaptured per night by BGM traps baited with Ifakara blend released by different odour-release devices in the semi-field system. BG-Cartridge and BG-Sachet refer to attractants infused inside microcapsules supplied by Biogents Company incased in a cylindrical plastic cartridge and plastic sachet, respectively; Nylon strips refer to batch of nylon strips where each strip is soaked in solution of a different constituent of the synthetic attractant; Control refers to an unbaited trap.

A total of 8000 mosquitoes were used in this experiment, of which 29.9% of the total were re-captured in the traps. The effect of the odour-dispensing mechanism was assessed using BGM trap baited with Ifakara lure dispensed from nylon strips, BG cartridge and BG sachets. Results of this experiment showed significantly higher mosquito catches when using any of the three dispensers, than the control (p < 0.001). The nightly median mosquito catches and the interquartile ranges are shown in Fig 7. The Tukey’s pair-wise comparison showed no significant difference between nylon strips and BG-Sachet (z = 0.15, p = 0.906), but a slight difference between BG-Sachet and BG-Cartridge (z = 0.15, p = 0.027) (Fig 6B). The model results shows that of the lure dispensers tested, BG-Cartridge attracted the most mosquitoes [RR = 96.03 (49.79–185.20), p<0.001)], followed by BG-Sachet [RR = 63.57 (32.92–122.76), p<0.001] and Nylon strips [RR = 57.46 (29.71–111.11), p<0.001] compared to the control.

## Discussion

The search for effective mosquito sampling tools remains a key priority for vector control practitioners and researchers. While the most appropriate trap may be different from place to place, and from vector species to species, it is important that all traps for large-scale field use are robust, easy to use, low-cost and have minimal need for replacement parts. The chemical nature of the attractants and attractant dispensing systems for use in these traps should also have similar attributes, but also be scalable, safe for human handling and readily available. This study describes the effectiveness of BG-Malaria (BGM) trap, an adaptation of BG-Sentinel (BGS) trap, as a sampling device for host-seeking *An*. *arabiensis* mosquitoes under semi-field conditions. Twice as many mosquitoes were caught by the BGM trap than by the BGS trap, which has previously been demonstrated as an effective trap for different mosquito species, including the malaria vector, *An*. *gambiae s*.*s* [46]. The BGS trap was originally designed for sampling *Aedes aegypti* mosquitoes [28], but because of its ease of use and demonstrated efficacy in a variety of field settings, it has become a good candidate for sampling malaria vectors [16, 46, 47] and is now widely used in research and surveillance settings.

In a field study conducted in Brazil [29], the BGM trap was demonstrated as being highly effective and nearly comparable to human landing catches, the most representative sampling strategy for human-biting mosquitoes [48]. By changing the BGS trap orientation and installing it upside down, 40 cm above the ground, it was observed that collections of the Brazilian malaria vector, *An*. *darlingi* were extremely increased [29]. In this current study, our results have demonstrated that similar modifications also work for laboratory-reared African malaria vector, *An*. *arabiensis*.

As with the BGS, the spacious interior of the BGM trap allows addition of a wide variety of attractants. Although it was originally proposed to be used with CO_2_ as bait and the BG-Lure, it has in different occasions been tested when baited with different attractants. In this study, we tested four synthetic attractants and three attractant dispensing methods. We observed that the BGM would work with a variety of attractants. Of all candidate lures tested, the Mbita blend (MB5) tended to be the most attractive, though the differences in attractiveness between lures was not always significant. To evaluate synthetic blends, CO_2_ was used as an effective mosquito attractant that also synergises other lures and activates mosquitoes [49, 50]. CO_2_ is routinely added to enhance mosquito responses in laboratory and field studies with odour blends [15, 16, 31, 47, 51]. The same procedure was adopted in this study and a trap baited with CO_2_ only was tested alongside an un-baited trap control to enable elucidation of any synergistic effects of CO_2_ with other lures.

Our findings reveal that traps baited with the synthetic blend, MB5, caught the highest number of mosquitoes, compared to the BG-Lure. However, the other attractants also performed well but there were no differences in the caches in traps baited with MB5, IB and CO_2_. Post hoc analyses suggest minor differences between the lure types, but a clear differences between any of the lures and the controls. Since we found only a minor synergy between the CO_2_ and the MB5 and IB attractants. This finding suggests that, for purposes of sampling the east African malaria vectors, BGM can be used with a variety of mosquito attractants and that even CO_2_ alone, in this case derived from yeast-molasses fermentation, may by itself be appropriate. Therefore, where the intention is purely surveillance, rather than vector control, BGM offers significant improvements over BG sentinel traps and can be fitted with any of the multiple synthetic odour lures available commercially or readily in local communities, such as CO_2_ derived from yeast-molasses fermentation.

The formulation of the MB5 contains ammonia, lactic acid, tetradecanoic acid, 3-methyl-1-butanol and butan-1-amine, and according to semi-field and field studies done in Kenya, it was shown to attract nearly as many mosquitoes as human subjects [38]. In addition, the MB5 was more attractive than IB, consistent with the findings of the present study. Even though the MB5 and IB share some candidate odorant constituents, the concentration of these chemical constituents may affect the responses of mosquitoes. A recent study demonstrated that attraction of mosquitoes to MB5 was concentration-dependent and that addition of 1-dodecanol to MB5 increased catches of female *An*. *gambiae* s.l. [36]. What is particularly important in this new study is that the MB5, being a far simpler blend of attractive constituents that IB, also tended to be the more attractive version. It is possible therefore, as concluded also by Okumu [31], that new odour mixtures can be developed with far superior attractiveness than any of the current blends.

Since their original tests, nylon strips are increasingly used as a matrix for dispensing attractant compounds [31, 33–36, 38]. In a comparison between LDPE and nylon strips, Mweresa *et al*. showed that releasing the IB from nylon strips caused a significantly higher attraction of *An*. *gambiae* [37]. Okumu *et al*., [33] also reported this effect. However, the current study indicated that BG-Cartridge attracted consistently higher proportions of laboratory *An*. *arabiensis*. Although, the BG-Cartridge presented the best results in *An*. *arabiensis* attraction in our study, these findings were obtained in tests done for short periods, unlike the nylon strips that have been tested previously for long-term dispensation [34, 37]. A previous semi-field study done in western Kenya showed that nylon strips treated with IB remained attractive to host-seeking mosquitoes for up to 40 consecutive nights post-treatment [34]. These findings were extended up to one year in a western Kenya study [37], but apparently this last result was likely affected by bacteria that establish on strips over time producing additional compounds that attract host-seeking mosquitoes. Nonetheless, further studies about alternative and low-cost odour release devices considering the difference in release ratios of semio-chemicals are needed. Thus, the possible matrix should also be tested for their constant release and long-lasting residual activity on target mosquitoes.

One limitation of this study was that we did not compare the BGM to several other trap types, especially the human landing catches, currently considered the gold standard. However, we selected to compare the BGM against an already established outdoor mosquito sampling tool such as the BGS and did not consider it essential to compare it to HLC. Such studies could be done in the future. Our study instead was considered an initial assessment of the potential of BGM for sampling Afro-tropical malaria mosquitoes.

## Conclusion

Earlier studies had already demonstrated that the BGM trap was highly effective for trapping the Brazilian malaria vector, *An*. *darlingi* [29, 30]. Here, we conclude that this trap is also effective for capturing the African malaria vector, *An*. *arabiensis*. To ascertain whether BGM could be an effective representative field sampling tool for malaria mosquitoes, and possibly a substitute the human landing catch, additional tests in field settings in malaria endemic communities are recommended. Overall, we conclude that BGM has potential as an improved sampling tool for malaria vectors in Africa, and that its efficacy can be optimized by selecting appropriate odour baits and appropriate odour-dispensing systems.
